# Trends of Acute Pericarditis-Related Mortality in the United States from 1999 to 2023: An Observational Analysis

**DOI:** 10.1055/s-0046-1824467

**Published:** 2026-07-10

**Authors:** Jawad Basit, Shahzaib Ahmed, Hoor ul Ain, Fatima Hussain, Eeman Ahmad, Mushood Ahmed, Aniq Saleem, Muhammad Usman, Raheel Ahmed, M. Chadi Alraies

**Affiliations:** 1 Department of Medicine, Rawalpindi Medical University, Rawalpindi, Pakistan; 2 Department of Medicine, Fatima Memorial Hospital College of Medicine and Dentistry, Lahore, Pakistan; 3 Department of Medicine, Mohtarma Benazir Bhutto Shaheed Medical College, Mirpur (AJK), Pakistan; 4 Department of Hospital Medicine, University of Wisconsin, Madison, Wisconsin, United States; 5 National Heart and Lung Institute, Imperial College London, London, United Kingdom; 6 Detroit Medical Center, Wayne State University, Detroit, Michigan, United States

**Keywords:** pericarditis, acute pericarditis, mortality trends, epidemiology, sex disparaties, geographic disparaties, cardiovascular mortality

## Abstract

**Background:**

Acute pericarditis (AP) is the acute inflammation of the pericardium that may be associated with infectious or non-infectious etiologies. A comprehensive analytical review of the mortality trends associated with AP is essential in order to identify population groups that are at the highest risk of mortality.

**Methods:**

We extracted age-adjusted mortality rates (AAMRs) per 100,000 persons from the Centers for Disease Control and Prevention (CDC) Wide-ranging ONline Data for Epidemiologic Research (WONDER) database. Data were stratified according to sex, census regions, and urbanization. Changes in AAMRs were analyzed by calculating annual percentage change (APC) through Joinpoint regression.

**Results:**

A total of 4,706 deaths were reported among individuals with AP in the United States from 1999 to 2023. The AAMRs decreased considerably from 1999 to 2014 (APC: −4.48), followed by an increase till 2023 (APC: 5.67). Males had a higher overall AAMR (0.07) in comparison to females (0.04). The AAMRs for both males and females decreased initially (male 1999–2006 APC: −8.16; female 1999–2010 APC: −4.90), followed by an increase until 2023 (male 2006–2023 APC: 1.76; female 2010–2023 APC: 1.65). The West exhibited the highest AAMR (0.07), followed by the Midwest (0.06), the South (0.05), and the Northeast (0.04). Individuals residing in non-metropolitan areas exhibited a higher AAMR (0.06) compared to those residing in metropolitan areas (0.04). The AAMR for non-metropolitan areas remained stable (APC: −0.36), whereas that for metropolitan areas declined considerably (APC: −3.05).

**Conclusion:**

These findings highlight the need for the implementation of focused public health policies, equitable resource allocation, and improved cardiovascular health care facilities to mitigate the rising AP-related AAMRs in vulnerable populations, especially male residents of the West and Midwest census regions.

## Introduction

Acute pericarditis (AP) is an inflammatory condition affecting the pericardium, with most cases being idiopathic while the rest are attributed to infectious or non-infectious pathologies. 1 Common infectious causes include viral pathogens such as coxsackievirus, echovirus, and severe acute respiratory syndrome coronavirus 2 (SARS-CoV-2), while non-infectious causes include autoimmune diseases (e.g., systemic lupus erythematosus), malignancies, uremia, and post-cardiac injury syndromes (e.g., Dressler's syndrome). 1 Although most cases of this disease follow a benign self-limited course and respond well to conservative management with non-steroidal anti-inflammatory drugs (NSAIDs) and colchicine, complications such as cardiac tamponade and constrictive pericarditis can arise, requiring hospitalization. 2

Recent evidence suggests that coronavirus disease 2019 (COVID-19) is associated with an increased risk of pericarditis-related complications and mortality. 3 This might be due to an amplified immune response to the viral cytokine storm, as well as COVID-19-related pericardial effusion, which can impair cardiac function and lead to hemodynamic instability. Hypoxia and systemic inflammation further exacerbate organ dysfunction, increasing the risk of acute kidney injury (AKI). 4 5 Not only does primary SARS-CoV-2 infection appear to predispose patients to AP with higher mortality, but emerging evidence also suggests a possible association between COVID-19 mRNA vaccination and a small increase in the incidence of AP. 6 7 8 A study by Fairweather et al suggested a 15-fold increase in the incidence of myocarditis/pericarditis from the pre-COVID to the post-COVID era. 9

Limited data exist on the long-term trends of AP-related mortality, particularly when stratified by race, sex, urbanization, and geographic region. To address this gap, this study examined mortality trends in the United States utilizing CDC WONDER data from 1999 to 2023, with specific attention to differences between the pre-pandemic (1999–2020) and post-pandemic (2021–2023) periods. By analyzing these trends concerning key demographic and geographic factors, this study aims to identify populations at high risk of AP-related mortality and provide insights that can aid health care providers and policymakers in addressing disparities in access to health care more effectively on a national scale.

## Methods

### Study Design

Population data utilized to calculate the AAMRs were obtained from the U.S. Census Bureau as incorporated within the Centers for Disease Control and Prevention (CDC) Wide-ranging ONline Data for Epidemiologic Research (WONDER) database from 1999 to 2023 for AP-related mortality in the United States. 10 Death certificates listing AP as either an underlying or contributing cause of death were identified using the International Classification of Diseases, 10th Revision (ICD-10) coding system, which retrieved all certificates under the following codes: I30.0; I30.1; I30.8; I30.9. 11 This study utilized publicly available, de-identified data, and thus, approval from an institutional review board was not required. Our study adhered to the Strengthening the Reporting of Observational Studies in Epidemiology (STROBE) guidelines for reporting requirements. 12

### Data Extraction

The extracted dataset included demographic information (age, sex, race/ethnicity), geographic classifications (census regions, state-level data), and urbanization categories. Race and ethnicity were categorized as non-Hispanic (NH) White, NH Black or African American, Hispanic or Latino, NH American Indian or Alaskan Native, and NH Asian or Pacific Islander. Geographic regions were classified according to U.S. Census Bureau definitions (Northeast, Midwest, South, and West). The 2013 U.S. Census classification system was employed to distinguish between metropolitan and non-metropolitan populations. 13

Age-adjusted mortality rates (AAMRs) were calculated per 100,000 population and stratified by multiple variables, including sex, race/ethnicity, geographic region, urbanization level, and state. Due to unreliable data points, the AAMRs for Hispanic or Latino, NH American Indian or Alaskan Native, and NH Asian or Pacific Islander populations were excluded from trend analyses.

### Statistical Analysis

We employed the Joinpoint Regression Program to calculate annual percentage changes (APCs) with corresponding 95% confidence intervals (CIs). 14 This approach begins with a simple linear model and systematically adds joinpoints when statistically significant changes in trend are detected, utilizing the Monte Carlo permutation method for significance testing. This enabled us to identify distinct trend segments throughout the study period, providing detailed insights into mortality pattern changes across various demographic subgroups. Statistical significance was established using two-tailed *t* -tests, with *p* -values less than 0.05 considered significant.

## Results

**Fig. 1:**
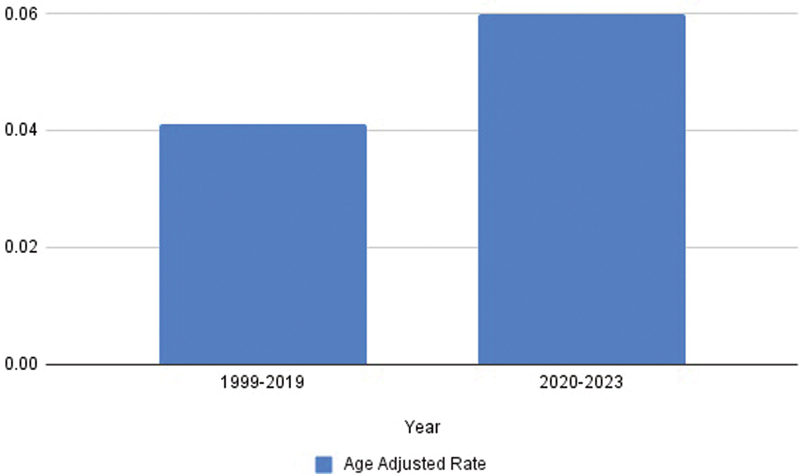
Comparison of age-adjusted mortality rates (AAMRs) per 100,000 individuals for acute pericarditis-related mortality before and during the COVID-19 pandemic. COVID-19, coronavirus disease 2019.

**Fig. 2:**
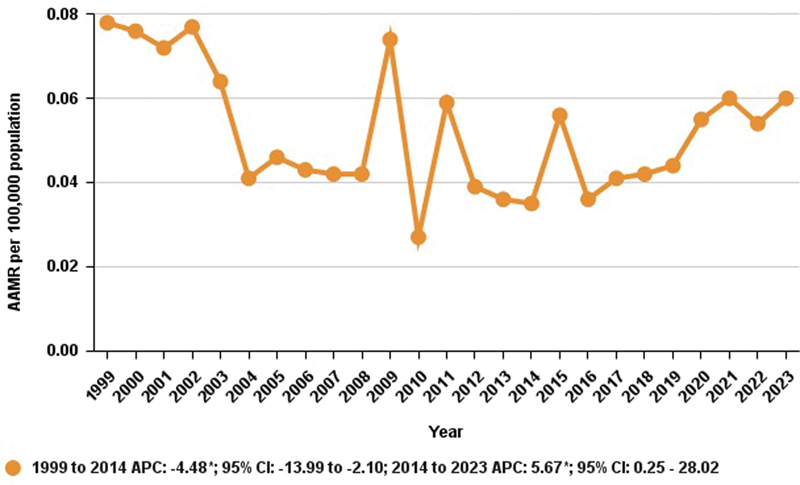
Trends in acute pericarditis-related mortality in the United States from 1999 to 2023. AAMR, age-adjusted mortality rate; APC, annual percentage change; CI, confidence interval.

From 1999 to 2023, a total of 4,706 deaths were reported among individuals with AP in the United States, with 14% occurring post-COVID-19 (2021–2023). The AAMRs per 100,000 individuals declined steadily from 1999 to 2014 (APC: −4.48; CI: −13.99 to −2.10) before shifting to a significant upward trend from 2014 to 2023 (APC: 5.67; CI: 0.25–28.02). The highest overall AAMR was recorded in 1999 (0.078), while between 2014 and 2023, the highest AAMRs were observed in 2021 and 2023 (0.060; Figs. 1 and 2 , Tables 1 and 2 , and Supplementary Table S1 [available in the online version only]).

**Table 1:** Demographic characteristics of acute pericarditis-related mortality in the United States from 1999 to 2023

Variable	Deaths	Population	AAMR (95% CI)
Overall			
1999–2020	4,003	6,746,356,647	0.041 (0.039–0.042)
2021–2023	703	1,000,096,197	0.06 (0.055–0.065)
Sex			
1999–2020			
Female	1,690	3,429,003,804	0.041 (0.039–0.043)
Male	2,313	3,317,352,843	0.074 (0.071–0.077)
2021–2023			
Female	270	504,678,502	0.042 (0.036–0.049)
Male	433	495,417,695	0.076 (0.068–0.083)
Race/Ethnicity			
1999–2020			
NH White	2,922	4,394,181,258	0.041 (0.039–0.043)
NH Black or African American	599	863,931,810	0.075 (0.069–0.082)
2021–2023			
NH White	493	588,491,981	0.06 (0.054–0.067)
NH Black or African American	95	126,242,095	0.066 (0.052–0.082)
Census region			
1999–2020			
Northeast	620	1,212,994,922	0.041 (0.037–0.045)
Midwest	982	1,466,121,214	0.049 (0.046–0.053)
South	1,283	2,497,818,081	0.036 (0.034–0.039)
West	1,118	1,569,422,430	0.074 (0.069–0.079)
2021–2023			
Northeast	72	171,183,761	0.024 (0.018–0.032)
Midwest	192	206,538,322	0.077 (0.065–0.089)
South	241	386,066,811	0.059 (0.05–0.067)
West	198	236,307,303	0.076 (0.064–0.087)
Urbanization			
1999–2020			
Urban	3,309	5,739,475,649	0.041 (0.039–0.043)
Rural	694	1,006,871,652	0.064 (0.058–0.069)

Abbreviations: AAMR, age-adjusted mortality rate; CI, confidence interval; NH, non-Hispanic.

The values in the population column represent the cumulative population estimates over the specified time intervals (1999–2020 and 2021–2023).

**Table 2:** Annual percentage changes and average annual percentage changes in acute pericarditis-related mortality in the United States from 1999 to 2023

Variable	Trend segment	Lower endpoint	Upper endpoint	APC (95% CI)	AAPC (95% CI)	*p* -Value
Entire cohort	1	1999	2014	−4.48 a (−13.99 to −2.10)	−0.79 (−2.73 to 0.96)	0.363127
	2	2014	2023	5.67 a (0.25 to 28.02)		
Sex						
Female	1	1999	2010	−4.90 a (−21.11 to −1.27)	−1.41 (−3.06 to 0.33)	0.113977
	2	2010	2023	1.65 (−1.40 to 19.42)		
Male	1	1999	2006	−8.16 a (−23.24 to −2.73)	−1.24 (−2.52 to 0.13)	0.070786
	2	2006	2023	1.76 a (0.24 to 5.86)		
Race/Ethnicity						
NH White	1	1999	2010	−5.39 a (−13.10 to −2.61)	−0.60 (−1.96 to 0.68)	0.309538
	2	2010	2023	3.64 a (1.04 to 9.54)		
NH Black or African American	1	1999	2001	−29.73 a (−42.46 to −1.76)	−3.81 a (−5.49 to −0.97)	0.008398
	2	2001	2023	−1.03 (−6.96 to 13.86)		
Census region						
Northeast	1	1999	2023	−2.67 a (−4.70 to −0.70)	−2.67 a (−4.70 to −0.70)	0.004399
Midwest	1	1999	2012	−5.02 a (−15.78 to −1.89)	−0.33 (−2.20 to 1.58)	0.731454
	2	2012	2023	5.53 a (1.16 to 21.87)		
South	1	1999	2013	−4.21 a (−8.75 to −1.96)	−0.17 (−1.39 to 1.07)	0.803439
	2	2013	2023	5.77 a (1.97 to 14.20)		
West	1	1999	2019	−2.74 a (−10.79 to −1.34)	−0.50 (−2.42 to 0.99)	0.474305
	2	2019	2023	11.50 (−0.95 to 41.59)		
Urbanization						
Urban	1	1999	2020	−3.05 a (−4.73 to −1.42)	−3.05 a (−4.73 to −1.42)	<0.000001
Rural	1	1999	2020	−0.36 (−2.58 to 1.89)	−0.36 (−2.58 to 1.89)	0.701460

Abbreviations: APC, annual percentage change; AAPC, average annual percentage change; CI, confidence interval; NH, non-Hispanic.

a Indicates that the APC or AAPC is significantly different from zero at the alpha level = 0.05.

### Trends by Sex

**Fig. 3:**
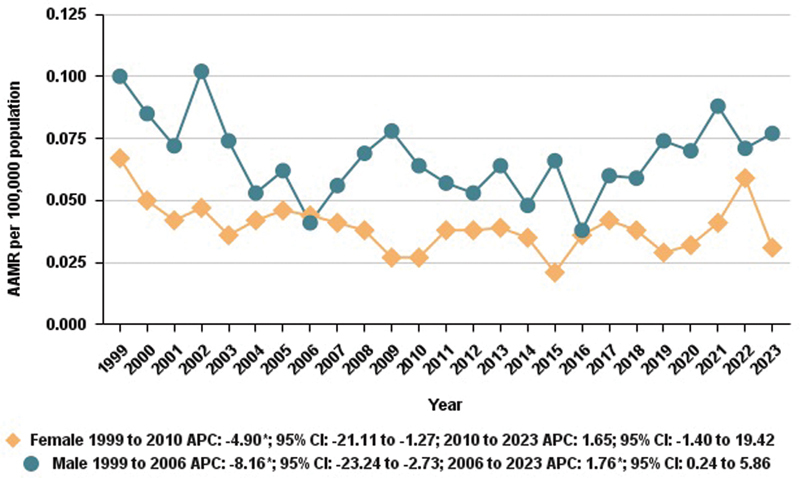
Trends in acute pericarditis-related mortality stratified by sex in the United States from 1999 to 2023. AAMR, age-adjusted mortality rate; APC, annual percentage change; CI, confidence interval.

Males had a significantly higher overall AAMR (1999–2023: 0.067; 1999–2020: 0.074; 2021–2023: 0.076) throughout both study periods compared to females, with both sexes showing a slight increase post-2021 (1999–2023: 0.039; 1999–2020: 0.041; 2021–2023: 0.042). The mortality rates for males declined considerably from 1999 to 2006 (APC: −8.16; CI: −23.24 to −2.73) before steadily rising until 2023 (APC: 1.76; CI: 0.24–5.86). Similarly, the AAMR for females followed a comparable pattern, decreasing from 1999 to 2010 (APC: −4.90; CI: −21.11 to −1.27), followed by a period of stabilization until 2023 (APC: 1.65; CI: −1.40 to 19.42; Fig. 3 , Tables 1 and 2 , and Supplementary Table S2 [available in the online version only]).

### Trends by Race

NH Black or African Americans reported a higher overall AAMR (0.075) compared to NH Whites (0.041) in the initial study period (1999–2020). From 2021 to 2023, the AAMRs of both races became comparable (NH Black or African Americans: 0.066; NH Whites: 0.060). From 1999 to 2001, the mortality rate for NH Black or African Americans showed a steep and significant decline (APC: −29.73; CI: −42.46 to −1.76) before gradually stabilizing until 2023 (APC: −1.03; CI: −6.96 to 13.86). NH Whites experienced a steady decline in AAMR from 1999 to 2010 (APC: −5.39; CI: −13.11 to −2.61), followed by a gradual rise until 2023 (APC: 3.64; CI: 1.04–9.54; Tables 1 and 2 , Supplementary Table S3 [available in the online version only]).

### Trends by Urbanization

**Fig. 4:**
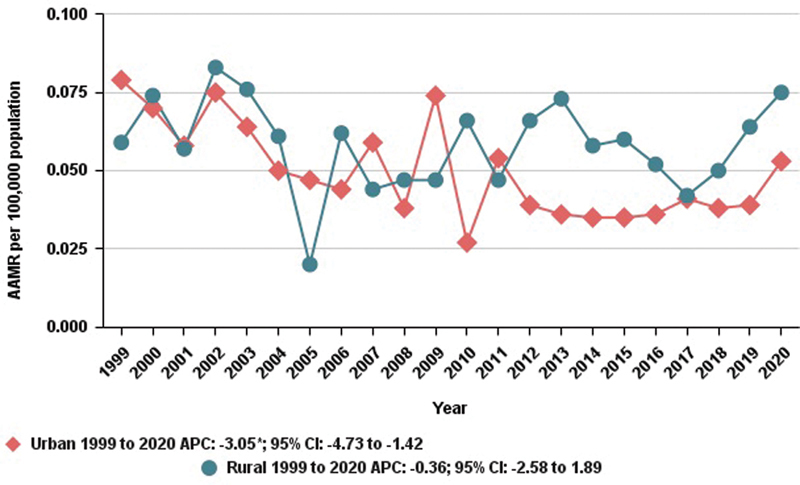
Trends in acute pericarditis-related mortality stratified by urbanization in the United States from 1999 to 2023. AAMR, age-adjusted mortality rate; APC, annual percentage change; CI, confidence interval.

From 1999 to 2020, non-metropolitan areas exhibited a higher AAMR (0.064) compared to individuals residing in metropolitan areas (0.041). The AAMR for non-metropolitan areas remained stable (APC: −0.36; CI: −2.58 to 1.89), whereas that for metropolitan areas declined considerably (APC: −3.05; CI: −4.73 to −1.42). Data for urbanization mortality trends were not available for 2021 to 2023 ( Fig. 4 , Tables 1 and 2 , and Supplementary Table S4 [available in the online version only]).

### Trends by Census Region

From 1999 to 2023, the West exhibited the highest AAMR (0.07), followed by the Midwest (0.06), the South (0.05), and the Northeast (0.04). However, between 1999 and 2020, the West had the highest AAMR (0.071), followed by the Midwest (0.049), the Northeast (0.041), and the South (0.036). From 2021 to 2023, AAMRs increased across all regions, with the Midwest (0.077) reporting the highest, followed closely by the West (0.076), the South (0.059), and the Northeast (0.024).

The West showed a slight decrease in AAMR from 1999 to 2019 (APC: −2.74; CI: −10.79 to −1.34), followed by a sharp but insignificant increase through 2023 (APC: 11.50; CI: −0.95 to 41.59). The Midwest and South followed similar trends, both experiencing declines until 2012 and 2013, respectively (Midwest APC: −5.03; CI: −15.78 to −1.89; South APC: −4.21; CI: −8.75 to −1.96), before rising significantly through 2023 (Midwest APC: 5.53; CI: 1.16–21.87; South APC: 5.77; CI: 1.97–14.20).

**Fig. 5:**
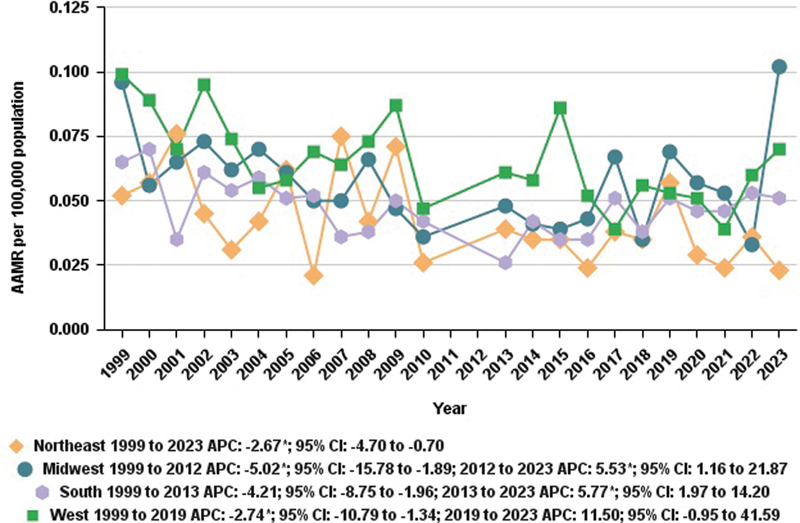
Trends in acute pericarditis-related mortality stratified by census region in the United States from 1999 to 2023. AAMR, age-adjusted mortality rate; APC, annual percentage change; CI, confidence interval.

In contrast, the Northeast exhibited a consistent linear decline in AAMR from 1999 to 2023 (APC: −2.67; CI: −4.70 to −0.70; Fig. 5 , Tables 1 and 2 , and Supplementary Table S5 [available in the online version only]).

### Trends by States

From 1999 to 2020, the states with the highest overall AAMRs were Washington (0.089), Oklahoma (0.080), Michigan (0.077), and Colorado (0.070), while the lowest AAMRs were observed in Alabama (0.013), Mississippi (0.013), and Virginia (0.014). From 2021 to 2023, Indiana (0.166), Colorado (0.146), and Michigan (0.12) had the highest overall AAMRs ( Supplementary Table S6 [available in the online version only]).

## Discussion

Our analysis revealed a steady decline in AAMR from 1999 to 2014, followed by a significant increase through 2023, with the highest AAMR recorded in 1999. Subsequent peaks were noted in 2021 and 2023, with 14% of all AP-related deaths occurring after 2021. Males reported consistently higher AAMRs than females throughout the study period, with both groups experiencing a slight increase post-2021. Racial disparities were also evident, as NH Black or African American individuals had higher AAMRs than NH Whites before 2021; however, post-2021, these rates became comparable. Geographically, non-metropolitan areas reported higher AAMRs than metropolitan areas from 1999 to 2020, while the Midwest and West had the highest overall mortality rates. From 2021 onwards, the Southern region also noted an increase in mortality, ranking third overall.

The pre-2014 decline in AP-related mortality can be attributed to several key factors, including improved treatment practices. In particular, colchicine has proven efficacious in reducing recurrent pericarditis in AP patients (or further recurrence after the first episode) in several major trials, including CORE (2005), ICAP (2013), CORP (2011), and CORP-2 (2014). 15 16 17 18 19 These findings have led to colchicine becoming a recommended treatment alongside NSAIDs in guidelines produced by the European Society of Cardiology (ESC) and approved by the American College of Cardiology (ACC) for the diagnosis and management of AP. 20 Subsequently, the mortality rates were observed to escalate after 2014. These results are reinforced by another study by Khan et al, analyzing the pericardial disease-associated mortality trends. 21 This surge might be attributed to the aging U.S. population, together with an increase in antiviral-resistant influenza virus specimens during that period. 22 23 This increase in resistant virus strains may have adversely affected patient outcomes because most idiopathic causes of pericarditis are viral in origin. 24

There has been an uptick in AP-related mortality with recent peaks in 2021 and 2023, which appears linked to the COVID-19 pandemic—the basis for stratifying our analysis into the periods 1999–2020 and 2021–2023. 3 The higher mortality rates in AP patients with COVID-19 compared to those without may be associated with an amplified immune response due to viral cytokine storms and pericardial effusion leading to hemodynamic instability. Systemic inflammation and hypoxia may further worsen multiorgan dysfunction, especially AKI. 3 4 5 Not only has pericarditis emerged as a major clinical manifestation of COVID-19, especially in adolescent males, but COVID-19-associated pericarditis has been linked to poorer clinical outcomes and worse cardiovascular sequelae as compared to AP alone. 25 26 27 This may explain the surge in deaths due to AP following the COVID-19 pandemic. Additionally, one of the serious adverse events following the administration of COVID-19 mRNA vaccines (Pfizer and Moderna) has been reported to be myocardial and pericardial inflammation. Interestingly, these cases have been observed in young adult males, the same cohort that has shown rising AP-associated mortality rates in our analysis. 28 29 30 31 However, future studies are warranted to establish an undisputed association between the vaccine administration and increasing mortality due to AP.

Our study also revealed prominent racial disparities pre-2021, with NH Black or African American individuals having a consistently higher (approximately twofold) mortality rate than their NH White counterparts. During the pandemic, the AAMRs for both races became more comparable, though NH Black or African American individuals still maintained a slightly higher rate. This is consistent with previous studies where Black individuals had higher all-cause mortality rates than White individuals. 32 This mortality pattern among Black patients may be due to higher incidences of chronic kidney disease in Black patients hospitalized for AP, though this requires further research to be substantiated. 33 Extrapolations can also be established from broader cardiovascular mortality disparities, as Black adults often have poorer access to health care and are more likely to receive care at subpar-quality facilities. 34 35 36 Post-2021, the mortality rates for both races have converged; the increase in White mortality appears associated with the generally higher incidence of mortality in AP patients with COVID-19, as discussed above.

Males suffering from AP were found to have almost double the mortality rates as females. Numerous studies have documented an increased risk of incidence and hospitalization for AP in males, 37 38 while some studies show a preference for females. 39 However, sex has not been proven to be an independent factor for in-hospital mortality, unlike increasing age or severe coinfection. 40 The higher mortality in non-metropolitan areas could be linked to limited health care access, lack of health insurance, poverty, and the presence of risk factors that lead to worse outcomes, such as smoking, hypertension, and obesity. 41 42 43 Similar factors may explain the rates in western regions. 44

## Limitations

First, these data are dependent on ICD codes, which can be misclassified, especially in cases where deaths occur outside of hospitals. Potential inaccuracies in death certification, variability in coding practices, diagnostic uncertainty, and temporal changes in reporting may lead to incorrect attribution of AP as either the underlying or contributing cause of death, resulting in over- or underestimation of mortality rates. Second, there is a lack of data on comorbidities and other determinants of health, such as the severity of illness or treatment received, which may affect overall mortality. Third, the unavailability of data on the incidence of AP during the study duration is a major limitation. We are unable to ascertain whether the rise in mortality rates truly reflects an increase in case fatality or higher disease occurrence, or is attributable to a combination of both. Future studies analyzing both the incidence and mortality data are warranted to better elucidate these trends. Despite our findings, a causal relationship between COVID-19 and AP mortality cannot be established.

## Conclusion

AP-related mortality in the United States has undergone a gradual decline from 1999 to 2014 before a significant increase was noted from 2014 through to 2023, with sharp upticks in the post-pandemic era. The highest AAMRs were observed in males, NH Black or African Americans, and those belonging to non-metropolitan or West and Midwest census regions. Targeted interventions and policies should be developed to better counter the rising AP-related mortality rates and reduce disease burden, especially among susceptible subgroups. Public health strategies aimed at improving cardiovascular care access for marginalized communities, equitable resource allocations, and possible cardiovascular screening in COVID-19-affected or vaccinated individuals should be drafted and implemented with possible extrapolation of these practices internationally.
