# Supplementary material for: Trends of Acute Pericarditis-Related Mortality in the United States from 1999 to 2023: An Observational Analysis
Source: Avicenna J Med. 2026 Jul 10;16(2):77–85. doi: 10.1055/s-0046-1824467 (PMC13354508; doi:10.1055/s-0046-1824467)
Supplement: Supplementary file 1 — Supplementary Material [file 10-1055-s-0046-1824467-s250163.pdf]

Supplementary File

Supplementary Table S1 Trends in acute pericarditis-related mortality in the United States from 1999 to 2023

Year	Age-adjusted rate	Age-adjusted rate Lower 95% confidence interval	Age-adjusted rate Upper 95% confidence interval	Age-adjusted rate Standard error
1999	0.078	0.067	0.089	0.006
2000	0.076	0.065	0.088	0.006
2001	0.072	0.061	0.084	0.006
2002	0.077	0.066	0.088	0.006
2003	0.064	0.053	0.074	0.005
2004	0.041	0.034	0.048	0.003
2005	0.046	0.038	0.054	0.004
2006	0.043	0.036	0.05	0.004
2007	0.042	0.035	0.049	0.004
2008	0.042	0.035	0.05	0.004
2009	0.074	0.063	0.085	0.006
2010	0.027	0.022	0.033	0.003
2011	0.059	0.048	0.069	0.005
2012	0.039	0.033	0.046	0.003
2013	0.036	0.03	0.042	0.003
2014	0.035	0.029	0.041	0.003
2015	0.056	0.046	0.066	0.005
2016	0.036	0.03	0.043	0.003
2017	0.041	0.034	0.048	0.004
2018	0.042	0.036	0.049	0.003
2019	0.044	0.036	0.052	0.004
2020	0.055	0.046	0.064	0.005
2021	0.06	0.051	0.069	0.004
2022	0.054	0.046	0.062	0.004
2023	0.06	0.05	0.07	0.005

© 2026. The Author(s).

Supplementary Table S2 Trends in acute pericarditis-related mortality stratified by gender in the United States from 1999 to 2023

Gender	Year	Age-adjusted rate	Age-adjusted rate Lower 95% confidence interval	Age-adjusted rate Upper 95% confidence interval
Female	1999	0.067	0.052	0.084
Female	2000	0.05	0.039	0.064
Female	2001	0.042	0.032	0.055
Female	2002	0.047	0.037	0.06
Female	2003	0.036	0.026	0.05
Female	2004	0.042	0.032	0.055
Female	2005	0.046	0.035	0.06
Female	2006	0.044	0.033	0.058
Female	2007	0.041	0.032	0.052
Female	2008	0.038	0.029	0.049
Female	2009	0.027	0.021	0.036
Female	2010	0.027	0.02	0.037
Female	2011	0.038	0.028	0.049
Female	2012	0.038	0.028	0.05
Female	2013	0.039	0.029	0.052
Female	2014	0.035	0.027	0.045
Female	2015	0.021	0.015	0.029
Female	2016	0.036	0.028	0.047
Female	2017	0.042	0.033	0.054
Female	2018	0.038	0.029	0.049
Female	2019	0.029	0.022	0.038
Female	2020	0.032	0.024	0.039
Female	2021	0.041	0.031	0.053
Female	2022	0.059	0.045	0.075
Female	2023	0.031	0.023	0.039
Male	1999	0.1	0.082	0.119
Male	2000	0.085	0.069	0.102
Male	2001	0.072	0.058	0.089
Male	2002	0.102	0.083	0.12
Male	2003	0.074	0.059	0.089
Male	2004	0.053	0.041	0.066
Male	2005	0.062	0.049	0.078
Male	2006	0.041	0.032	0.052
Male	2007	0.056	0.043	0.068
Male	2008	0.069	0.055	0.083
Male	2009	0.078	0.063	0.093
Male	2010	0.064	0.051	0.081
Male	2011	0.057	0.044	0.072
Male	2012	0.053	0.042	0.067
Male	2013	0.064	0.05	0.078
Male	2014	0.048	0.038	0.06
Male	2015	0.066	0.053	0.081

(*Continued*)

 © 2026. The Author(s).

Supplementary Table S2 (*Continued*)

Gender	Year	Age-adjusted rate	Age-adjusted rate Lower 95% confidence interval	Age-adjusted rate Upper 95% confidence interval
Male	2016	0.038	0.028	0.05
Male	2017	0.06	0.047	0.075
Male	2018	0.059	0.047	0.071
Male	2019	0.074	0.06	0.088
Male	2020	0.07	0.055	0.084
Male	2021	0.088	0.073	0.102
Male	2022	0.071	0.059	0.084
Male	2023	0.077	0.062	0.091

Supplementary Table S3 Trends in acute pericarditis-related mortality stratified by race in the United States from 1999 to 2023

Race	Year	Age-adjusted rate	Age-adjusted rate Lower 95% confidence interval	Age-adjusted rate Upper 95% confidence interval
White	1999	0.071	0.059	0.083
White	2000	0.056	0.046	0.066
White	2001	0.064	0.052	0.076
White	2002	0.059	0.049	0.069
White	2003	0.065	0.053	0.078
White	2004	0.045	0.036	0.054
White	2005	0.046	0.037	0.055
White	2006	0.043	0.035	0.051
White	2007	0.041	0.033	0.048
White	2008	0.038	0.031	0.045
White	2009	0.061	0.049	0.073
White	2010	0.027	0.021	0.034
White	2011	0.041	0.032	0.049
White	2012	0.038	0.03	0.046
White	2013	0.041	0.033	0.049
White	2014	0.041	0.033	0.05
White	2015	0.057	0.045	0.069
White	2016	0.038	0.03	0.046
White	2017	0.041	0.033	0.049
White	2018	0.044	0.035	0.052
White	2019	0.057	0.045	0.07
White	2020	0.055	0.043	0.066
White	2021	0.067	0.055	0.078
White	2022	0.054	0.044	0.065
White	2023	0.053	0.042	0.064
Black or African American	1999	0.178	0.129	0.238
Black or African American	2000	0.115	0.078	0.162
Black or African American	2001	0.056	0.033	0.09
Black or African American	2002	0.107	0.072	0.154
Black or African American	2003	0.072	0.048	0.105

 © 2026. The Author(s).

Supplementary Table S3 (*Continued*)

Race	Year	Age-adjusted rate	Age-adjusted rate Lower 95% confidence interval	Age-adjusted rate Upper 95% confidence interval
Black or African American	2004	0.106	0.071	0.151
Black or African American	2005	0.093	0.062	0.135
Black or African American	2006	0.061	0.036	0.098
Black or African American	2007	0.072	0.045	0.111
Black or African American	2008	0.063	0.037	0.101
Black or African American	2009	0.119	0.084	0.164
Black or African American	2010	0.065	0.039	0.102
Black or African American	2012	0.086	0.055	0.126
Black or African American	2013	0.055	0.033	0.086
Black or African American	2014	0.063	0.039	0.096
Black or African American	2017	0.054	0.032	0.086
Black or African American	2018	0.086	0.058	0.122
Black or African American	2019	0.087	0.058	0.124
Black or African American	2020	0.069	0.044	0.103
Black or African American	2021	0.048	0.031	0.072
Black or African American	2022	0.072	0.045	0.109
Black or African American	2023	0.076	0.052	0.107

Supplementary Table S4 Trends in acute pericarditis-related mortality stratified by urbanization in the United States from 1999 to 2020

Year	Urbanization	Age-adjusted rate	Age-adjusted rate Lower 95% confidence interval	Age-adjusted rate Upper 95% confidence interval
1999	Urban	0.079	0.067	0.091
2000	Urban	0.07	0.058	0.081
2001	Urban	0.058	0.047	0.068
2002	Urban	0.075	0.063	0.088
2003	Urban	0.064	0.052	0.075
2004	Urban	0.05	0.041	0.058
2005	Urban	0.047	0.038	0.057
2006	Urban	0.044	0.036	0.053
2007	Urban	0.059	0.048	0.069
2008	Urban	0.038	0.031	0.045
2009	Urban	0.074	0.062	0.086
2010	Urban	0.027	0.021	0.033
2011	Urban	0.054	0.044	0.064
2012	Urban	0.039	0.032	0.047
2013	Urban	0.036	0.03	0.043
2014	Urban	0.035	0.028	0.041
2015	Urban	0.035	0.028	0.042
2016	Urban	0.036	0.029	0.044
2017	Urban	0.041	0.033	0.048
2018	Urban	0.038	0.032	0.044

(*Continued*)

 © 2026. The Author(s).

Supplementary Table S4 (*Continued*)

Year	Urbanization	Age-adjusted rate	Age-adjusted rate Lower 95% confidence interval	Age-adjusted rate Upper 95% confidence interval
2019	Urban	0.039	0.032	0.047
2020	Urban	0.053	0.044	0.063
1999	Rural	0.059	0.039	0.085
2000	Rural	0.074	0.05	0.107
2001	Rural	0.057	0.038	0.083
2002	Rural	0.083	0.059	0.114
2003	Rural	0.076	0.05	0.111
2004	Rural	0.061	0.038	0.093
2005	Rural	0.02	0.011	0.033
2006	Rural	0.062	0.041	0.09
2007	Rural	0.044	0.029	0.063
2008	Rural	0.047	0.03	0.07
2009	Rural	0.047	0.031	0.067
2010	Rural	0.066	0.04	0.103
2011	Rural	0.047	0.026	0.079
2012	Rural	0.066	0.044	0.095
2013	Rural	0.073	0.048	0.107
2014	Rural	0.058	0.038	0.083
2015	Rural	0.06	0.039	0.088
2016	Rural	0.052	0.035	0.074
2017	Rural	0.042	0.026	0.066
2018	Rural	0.05	0.029	0.079
2019	Rural	0.064	0.041	0.095
2020	Rural	0.075	0.049	0.11

Supplementary Table S5 Trends in acute pericarditis-related mortality stratified by census region in the United States from 1999 to 2023

Census region	Year	Age-adjusted rate	Age-adjusted rate Lower 95% confidence interval	Age-adjusted rate Upper 95% confidence interval
Census region 1				
Northeast	1999	0.052	0.034	0.076
Northeast	2000	0.057	0.039	0.081
Northeast	2001	0.076	0.053	0.106
Northeast	2002	0.045	0.028	0.068
Northeast	2003	0.031	0.018	0.049
Northeast	2004	0.042	0.025	0.066
Northeast	2005	0.062	0.041	0.09
Northeast	2006	0.021	0.012	0.035
Northeast	2007	0.075	0.052	0.104
Northeast	2008	0.042	0.027	0.064
Northeast	2009	0.071	0.05	0.098
Northeast	2010	0.026	0.014	0.043

 © 2026. The Author(s).

Supplementary Table S5 (*Continued*)

Census region	Year	Age-adjusted rate	Age-adjusted rate Lower 95% confidence interval	Age-adjusted rate Upper 95% confidence interval
Northeast	2013	0.039	0.023	0.062
Northeast	2014	0.035	0.023	0.051
Northeast	2015	0.035	0.021	0.054
Northeast	2016	0.024	0.015	0.038
Northeast	2017	0.038	0.023	0.058
Northeast	2018	0.035	0.021	0.055
Northeast	2019	0.057	0.038	0.082
Northeast	2020	0.029	0.019	0.043
Northeast	2021	0.024	0.014	0.039
Northeast	2022	0.036	0.022	0.056
Northeast	2023	0.023	0.013	0.038
Census region 2				
Midwest	1999	0.096	0.074	0.122
Midwest	2000	0.056	0.039	0.078
Midwest	2001	0.065	0.045	0.09
Midwest	2002	0.073	0.054	0.098
Midwest	2003	0.062	0.043	0.087
Midwest	2004	0.07	0.051	0.093
Midwest	2005	0.061	0.043	0.084
Midwest	2006	0.05	0.035	0.068
Midwest	2007	0.05	0.035	0.068
Midwest	2008	0.066	0.046	0.091
Midwest	2009	0.047	0.032	0.069
Midwest	2010	0.036	0.023	0.055
Midwest	2011	0.048	0.034	0.065
Midwest	2012	0.041	0.028	0.058
Midwest	2013	0.039	0.026	0.058
Midwest	2014	0.043	0.03	0.059
Midwest	2015	0.067	0.047	0.092
Midwest	2016	0.035	0.022	0.052
Midwest	2017	0.069	0.048	0.095
Midwest	2018	0.057	0.039	0.079
Midwest	2019	0.053	0.038	0.073
Midwest	2020	0.033	0.023	0.047
Midwest	2021	0.102	0.079	0.129
Midwest	2022	0.074	0.056	0.097
Midwest	2023	0.058	0.042	0.08
Census region 3				
South	1999	0.065	0.049	0.086
South	2000	0.07	0.054	0.089
South	2001	0.035	0.023	0.05
South	2002	0.061	0.047	0.078
South	2003	0.054	0.038	0.074

(*Continued*)

 © 2026. The Author(s).

Supplementary Table S5 (*Continued*)

Census region	Year	Age-adjusted rate	Age-adjusted rate Lower 95% confidence interval	Age-adjusted rate Upper 95% confidence interval
South	2004	0.059	0.043	0.078
South	2005	0.051	0.037	0.07
South	2006	0.052	0.038	0.071
South	2007	0.036	0.024	0.052
South	2008	0.038	0.027	0.053
South	2009	0.05	0.037	0.065
South	2010	0.042	0.029	0.059
South	2011	0.026	0.018	0.036
South	2012	0.042	0.03	0.058
South	2013	0.035	0.025	0.048
South	2014	0.035	0.025	0.048
South	2015	0.051	0.037	0.069
South	2016	0.038	0.028	0.05
South	2017	0.051	0.037	0.068
South	2018	0.046	0.035	0.059
South	2019	0.046	0.034	0.06
South	2020	0.053	0.04	0.069
South	2021	0.051	0.039	0.067
South	2022	0.057	0.044	0.074
South	2023	0.069	0.053	0.089
Census region 4				
West	1999	0.099	0.074	0.131
West	2000	0.089	0.066	0.118
West	2001	0.07	0.051	0.093
West	2002	0.095	0.072	0.123
West	2003	0.074	0.055	0.098
West	2004	0.055	0.038	0.077
West	2005	0.058	0.04	0.081
West	2006	0.069	0.05	0.094
West	2007	0.064	0.046	0.086
West	2008	0.073	0.053	0.099
West	2009	0.087	0.065	0.113
West	2010	0.047	0.032	0.066
West	2011	0.061	0.045	0.082
West	2012	0.058	0.043	0.076
West	2013	0.086	0.064	0.114
West	2014	0.052	0.037	0.071
West	2015	0.039	0.027	0.055
West	2016	0.056	0.038	0.078
West	2017	0.053	0.038	0.071
West	2018	0.051	0.037	0.068
West	2019	0.039	0.026	0.057
West	2020	0.06	0.045	0.079

 © 2026. The Author(s).

Supplementary Table S5 (*Continued*)

Census region	Year	Age-adjusted rate	Age-adjusted rate Lower 95% confidence interval	Age-adjusted rate Upper 95% confidence interval
West	2021	0.07	0.052	0.092
West	2022	0.07	0.053	0.09
West	2023	0.069	0.051	0.091

Supplementary Table S6 Trends in acute pericarditis-related mortality stratified by states in the United States from 1999 to 2020

State	Deaths	Age-adjusted rate	Age-adjusted rate Lower 95% confidence interval	Age-adjusted rate Upper 95% confidence interval
Alabama	28	0.013	0.006	0.023
Arizona	85	0.047	0.036	0.061
Arkansas	24	0.032	0.019	0.051
California	617	0.062	0.057	0.068
Colorado	75	0.074	0.058	0.094
Connecticut	28	0.023	0.014	0.035
Delaware	13	Unreliable	0.023	0.082
District of Columbia	17	Unreliable	0.07	0.199
Florida	249	0.038	0.032	0.043
Georgia	85	0.035	0.026	0.045
Hawaii	19	Unreliable	0.032	0.087
Idaho	16	Unreliable	0.031	0.096
Illinois	140	0.041	0.033	0.049
Indiana	99	0.056	0.045	0.07
Iowa	50	0.071	0.05	0.098
Kansas	47	0.072	0.051	0.097
Kentucky	58	0.051	0.037	0.068
Louisiana	55	0.05	0.036	0.067
Maryland	55	0.037	0.027	0.051
Massachusetts	79	0.056	0.043	0.071
Michigan	190	0.077	0.065	0.089
Minnesota	77	0.051	0.039	0.065
Mississippi	20	0.013	0.005	0.026
Missouri	65	0.036	0.026	0.049
Montana	15	Unreliable	0.033	0.111
Nebraska	23	0.071	0.044	0.108
Nevada	29	0.036	0.022	0.056
New Hampshire	17	Unreliable	0.026	0.081
New Jersey	91	0.032	0.025	0.042
New Mexico	29	0.054	0.034	0.082
New York	216	0.041	0.035	0.047
North Carolina	91	0.039	0.03	0.05
North Dakota	10	Unreliable	0.031	0.142
Ohio	171	0.044	0.037	0.051
Oklahoma	68	0.08	0.06	0.104

(*Continued*)

 © 2026. The Author(s).

Supplementary Table S6 (*Continued*)

State	Deaths	Age-adjusted rate	Age-adjusted rate Lower 95% confidence interval	Age-adjusted rate Upper 95% confidence interval
Oregon	42	0.036	0.025	0.05
Pennsylvania	153	0.041	0.033	0.048
Rhode Island	15	Unreliable	0.036	0.107
South Carolina	67	0.062	0.047	0.082
South Dakota	16	Unreliable	0.04	0.135
Tennessee	81	0.036	0.028	0.047
Texas	298	0.05	0.044	0.057
Utah	26	0.052	0.033	0.078
Vermont	14	Unreliable	0.05	0.167
Virginia	50	0.014	0.009	0.021
Washington	147	0.089	0.074	0.104
West Virginia	24	0.058	0.034	0.092
Wisconsin	94	0.052	0.041	0.065
Wyoming	15	Unreliable	0.056	0.173

 © 2026. The Author(s).
